# Annotations

**Published:** 1888-11-17

**Authors:** 


### ANNOTATIONS.

Procession or Puddings?

THE new Lord Mayor last week diminished the ninth of November procession in order that a large number of poor people might for once be supplied with a good meal. It is not a bad thing that we should thus have put before us in a practical way the question as to which is best," processions for the proud," or "puddings for the poor." Processions are not always mere pageantry, nor are they necessarily pure waste of time and money. There are occasions when the heart of a nation or of a city is stirred to its depths by some unusual and moving event, and when the roused feelings insist upon some mode of public demonstration. A patriotic war declared, or an honourable peace proclaimed; the birth of an heir to the throne, or the death of a well-beloved ruler, or other great public servant; these are occasions on which joy or sorrow insists upon expression, and the fit expression is the great spectacle of a whole people moved and swayed by an impulse common to them all. But no one will contend that such occasions are very frequent. An event which is annual, like the coming of winter, loses its power to excite. The event may be in itself of no mean importance, and yet the mere regularity of its recurrence deprives it of spontaneous enthusiasm. It seems to us that an annual change of civic rulers in the City of London, though a circumstance of no small significance in itself, is not fitly celebrated by a procession of which the best that can be said is that it is an incongruous medley of a circus parade, a suburban funeral, and a Brobdignagian State ceremonial. By all means let the new Lord Mayor's formal installation be publicly recognised, by all means let the people have their holiday; but the gift of food to thousands of the hungry, or the gathering together of tens of thousands in athletic competitions in one of the parks—these or other similar modes of celebrating the anniversary would show no marks of anachronous incongruity, would cause nobody to indulge in derisive smiles, but would give both old and young, rich and poor, that practical gratification which results from seeing large numbers truly pleased and happy. For ourselves, we have much more faith in puddings and play for the poor than in pompous processions for the privileged and prosperous few.

What is a Lunatic?

WHAT is a lunatic? A person of unsound mind! And what is "a person of unsound mind?" A lunatic! Most true; but how much knowledge have we gained in asking and answering the questions? Few things better illustrate the absolute worthlessness and worse than worthlessness of "rough and ready methods" of dealing with human affairs than the history of certain persons who have suffered from attacks of insanity. A short time ago Mr. Alfred Hyman Louis, a barrister, sued the Marylebone Guardians, and Mr. William Rayner, their medical officer, for £20,000 damages for alleged wrongful imprisonment in a lunatic asylum. We need not stop to point out the absurdity of the amount of damages claimed. The sum was fixed probably on the well-known principle that if a very large demand is made, there may be at least an approximate result. What we wish to point out is the interesting but common circumstance, that though Mr. Louis was taken charge of as a lunatic on a doctor's certificate, and though he seems to have conducted himself so decidedly after the usual fashion of undoubted lunatics as to convince all who had him in charge of his undoubted insanity; yet he was perfectly conscious of what was going on around him, and was able to reproduce all the details of his case in evidence three years after the event. Studying the case impartially, one would be inclined to conclude that Mr. Louis was at one and the same time both a lunatic and not a lunatic, both sane and insane. But is not this the very essence and gist of almost every case of lunacy? And is it not the very rock on which persons untrained in dealing with lunacy cases always split? Is not this where judges and lawyers, applying what they regard as the methods of common sense and legal experience to facts which lie altogether beyond their experience, also fail to apprehend the real merits of cases, and often lend themselves to the most serious injustice? The average mind does not seem to be able to understand at all that a person may be both sane and insane at the very same time: sane as possible on some points, mad as possible on others. The want of this power of thoroughly realising the actual condition of an insane person, leads to all sorts of mistakes and not seldom to much brutal treatment of lunatics, as well as injustice to those who have charge of them. We are not among the number of those who believe in the infallibility of specialists, far from it; but, other things being equal, we hold that an expert of large experience is certainly more to be relied upon as a rule than persons who have had neither training nor experience.

A Veritable New Cure.

THERE are new cures innumerable published in every newspaper, but hardly one in fifty is of any more value than powdered bread crumbs. The first feeling excited in the experienced medical mind by the announcement of a new cure is one of suspicion. Some doctor wants to compass a reputation by a short cut; some designing quack is intent upon making a fortune without working for it. The very latest thing proposed is the "Slöjd cure." It is proposed by a lady—Miss Caton, and it emanates from the small and not very famous town of Wisbech. Either of these circumstances is sufficient to condemn the proposal in many medical minds. Can any medical discovery be made out of London? Can any non-medical person be permitted so much as to express an opinion on medical questions? It is not to be thought of! Miss Caton, however, has ventured to suggest the "Slöjd cure" for such diseases as "dyspepsia, hysteria, etc.," and, notwithstanding the daring temerity of her suggestion, there is every reason to believe that she still survives. When such things happen it is plain that England, as a country, and the nineteenth century as a century, are no longer worth living in. But what is the "Slöjd cure!" The answer will relieve the professional mind! It is not a physic. The word itself looks as if it might have something to do with cricket; and, as a matter of fact, it has. Dr. W. G. Grace will doubtless be canonised and made the patron saint of the new cure. Miss Caton says the word "Slöjd" is a lineal descendent of "Slog," and that "Slog" means "skilful," "dexterous," &c. Mr. Grace, when in his best trim, is skilful and dexterous in an unrivalled degree. His "slogging" is a wonder to behold. The "cure" consists in the learning and practice of *Slöjd Carpentry.* This kind of carpentry is taught the ordinary school children at Nääs in Sweden. So far as we can ascertain from the report of Miss Caton's lecture, the girls learn it as well as the boys. Miss Caton herself went through a proper course of training, ending by the making, with her own hands, of a five o'clock tea-table. The system consists in the making of a graduated series of models from other models. There are several series of such models, the largest containing fifty; and in the full course of fifty models, the operater learns to use with more or less dexterity, no fewer than 187 tools. The instruction is not given by artizans, but by trained and educated teachers; one principal object being to train the children to exactness, and artizans are often much wanting in strict exactness. The work is said to be not only highly valuable in the development of muscular and vital energy, but also extremely interesting and even fascinating to the pupils. Miss Caton's principal object in her Wisbech lecture, was to commend the system as a valuable means of physical and mental training for the young. Her mention of it as a cure for dyspepsia and hysteria was a mere by-thought. There is no doubt, however, that even as a "cure," the "Slöjd" system would be infinitely more valuable than many much-vaunted new remedies. Concerning its use in education, Mr. Ruskin's opinion, quoted by Miss Caton, may fitly close this notice: "It would be part of my scheme of physical education," says the sole surviver of our two latter-day prophets, "that every youth in the state, from the King's son downwards, should learn to do something finely and thoroughly with his hands, so as to let him know what *touch* meant, and what stout craftmanship meant, and to inform him of many things besides which no man can learn but by some severely accurate discipline in *doing.* Let him once learn to take a straight shaving off a plank, or draw a fine curve without faltering, or lay a brick level in its mortar, and he has learned a multitude of other matter which no lips of man can ever teach him."

